# Supplementary figures and images for: Inferring parameters of cancer evolution in chronic lymphocytic leukemia
Source: PLoS Comput Biol. 2022 Nov 4;18(11):e1010677. doi: 10.1371/journal.pcbi.1010677 (PMC9668150; doi:10.1371/journal.pcbi.1010677)

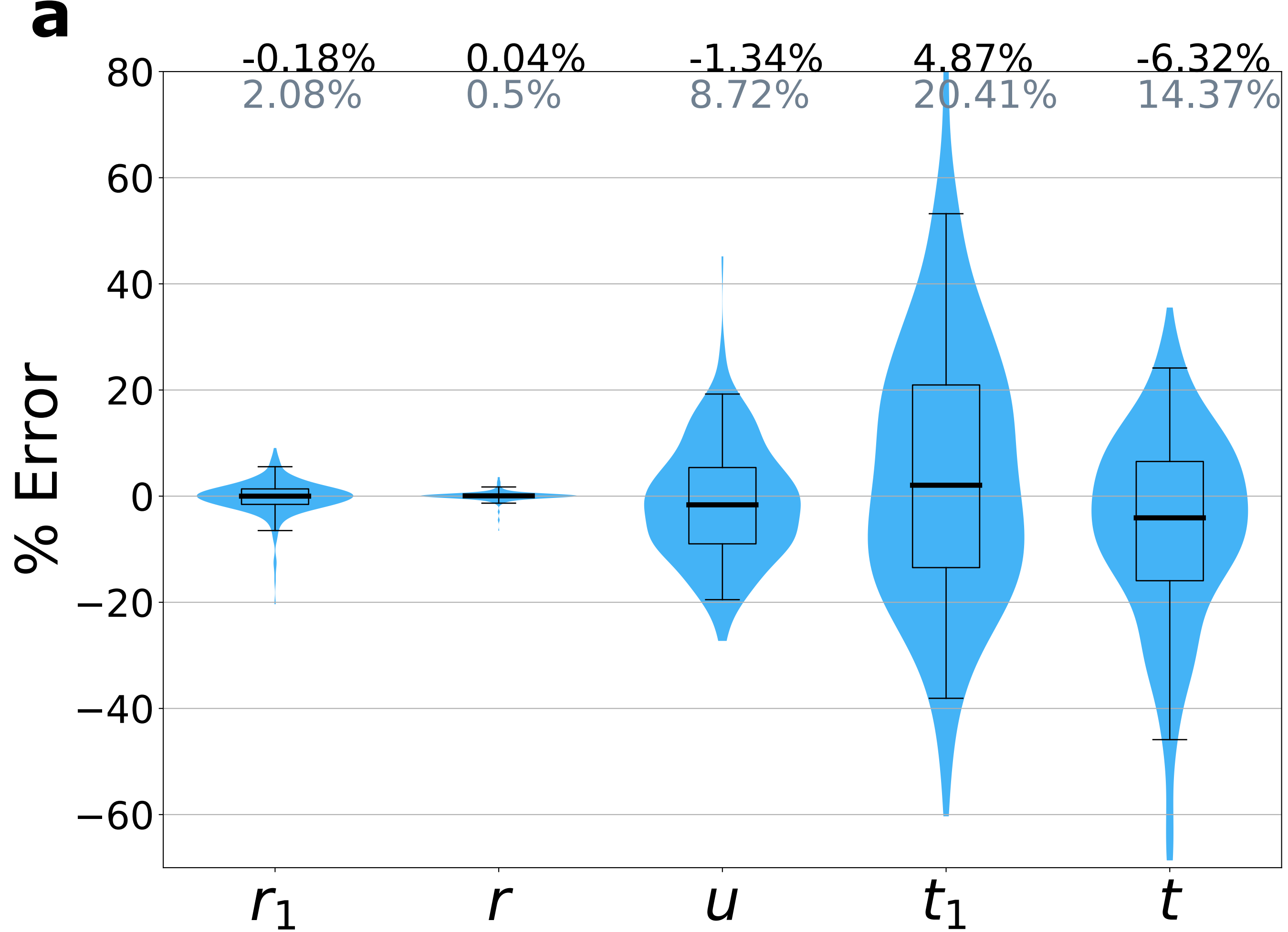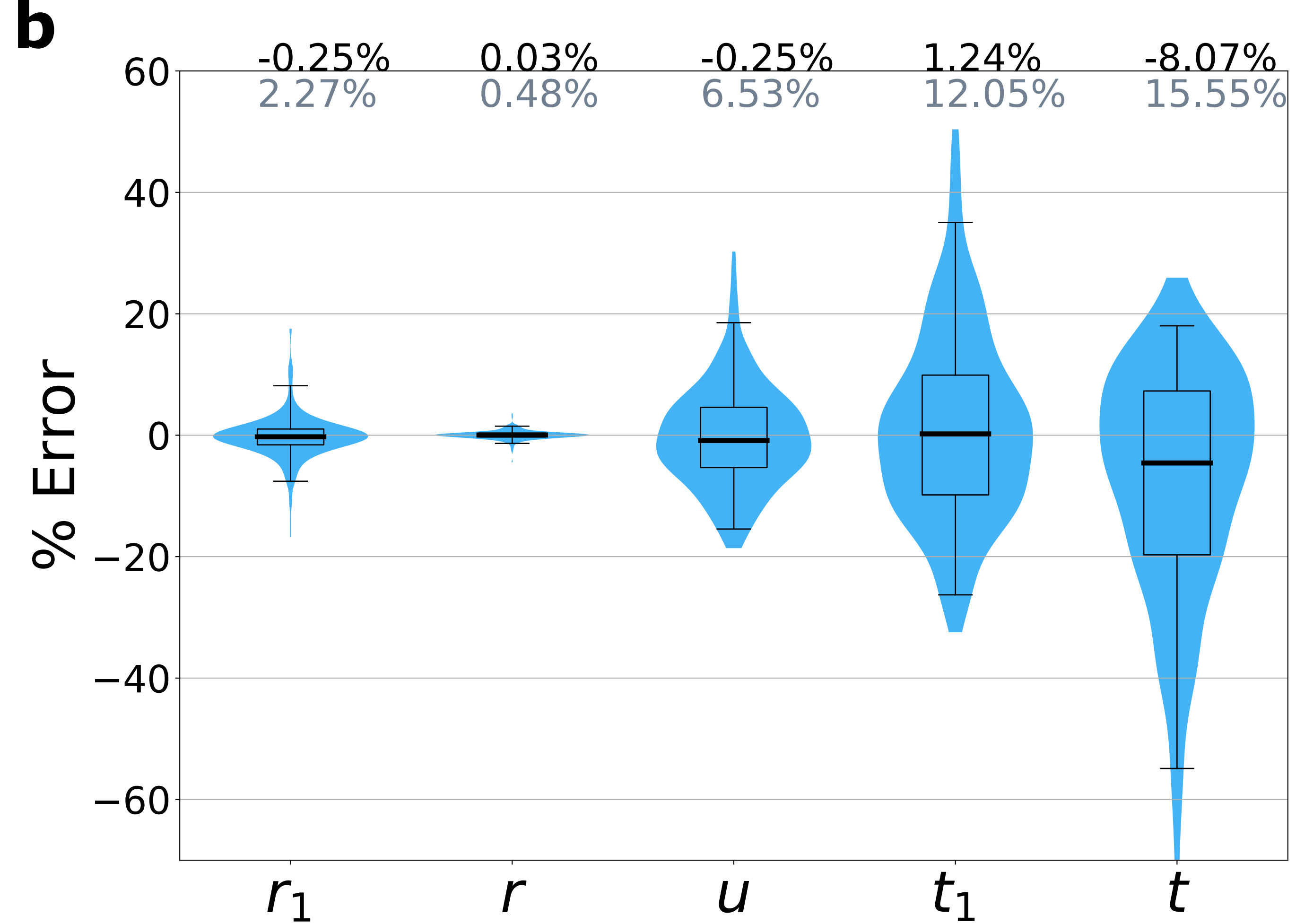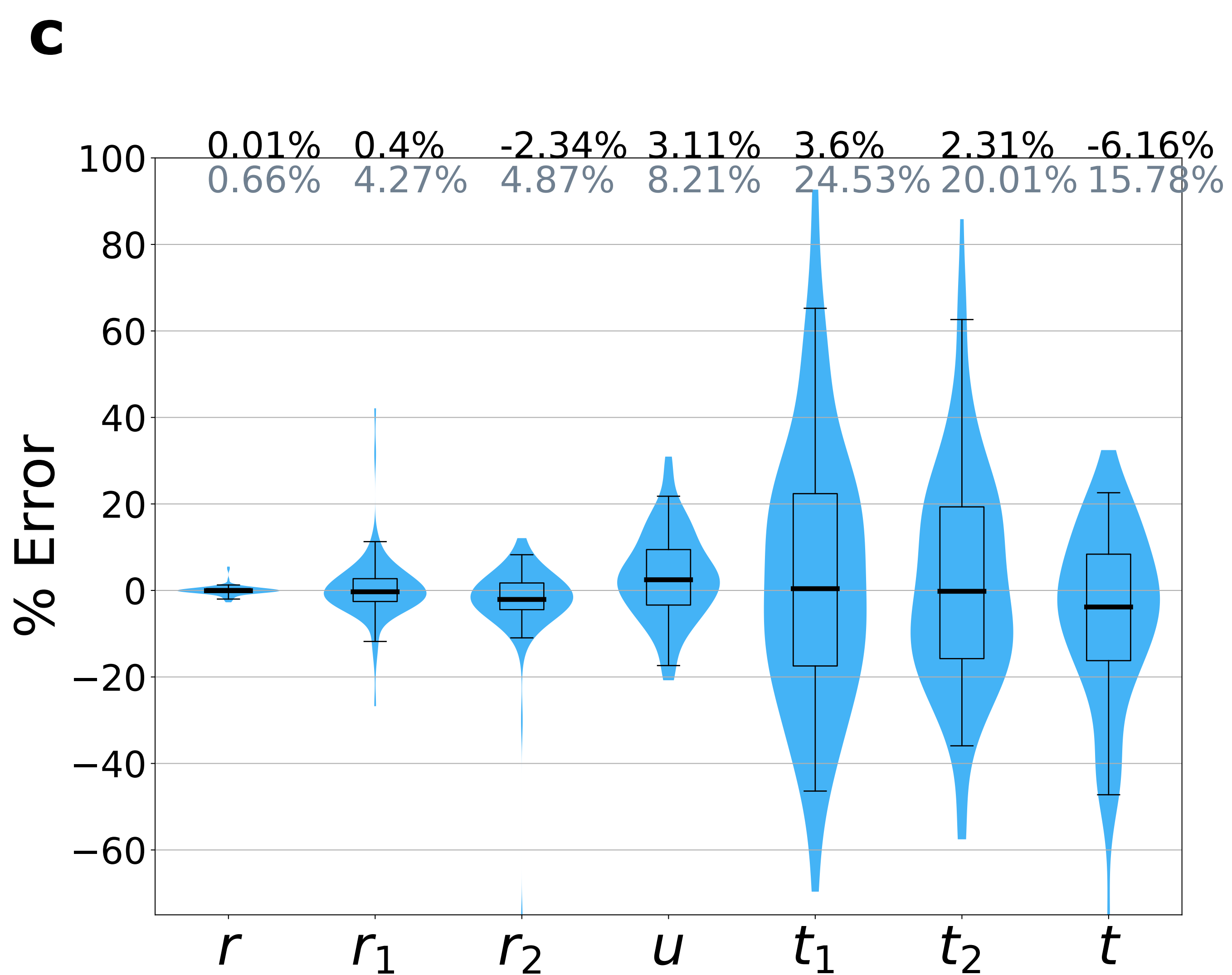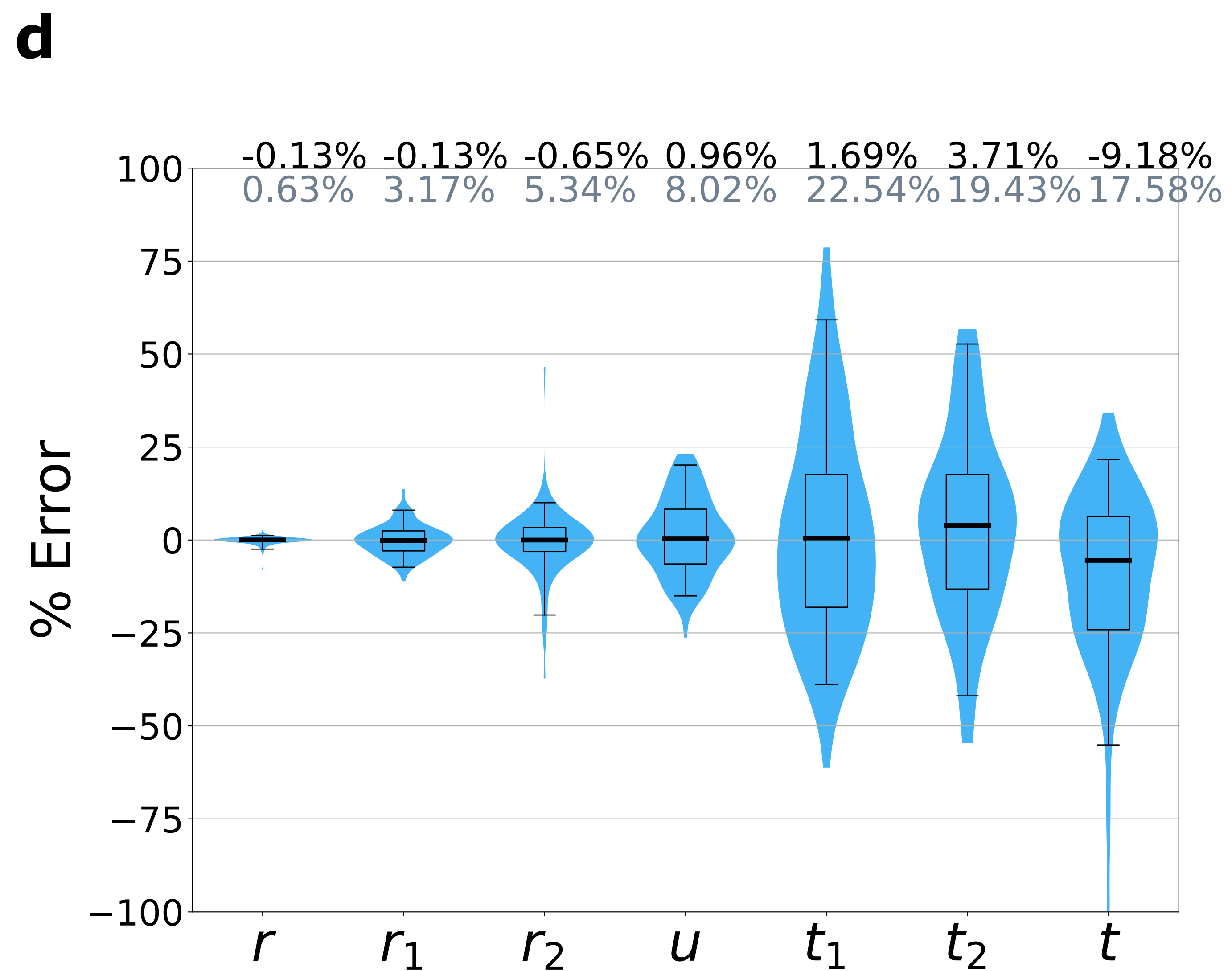

Supplement: S1 Fig — Accuracy of parameter inferences for Monte Carlo simulation of tumor with no cell death for (a) single driver subclone with mutation rate u = 1, (b) single driver subclone with u = 10, (c) two nested subclones with u = 1, and (d) two sibling subclones with u = 1. Mean percent error (MPEs) are the black numbers above the plots, and mean absolute percent errors (MAPEs) are the grey numbers below the MPEs. Boxes contain 25th-75th quartiles, with median indicated by thick horizontal black line. Whiskers of boxplots indicate 2.5 and 97.5 percentiles. Violins are smoothed density estimates of the percent error datapoints. Complete parameter values and number of runs are included in S1 Table. (PDF) [file pcbi.1010677.s001.pdf]

**a**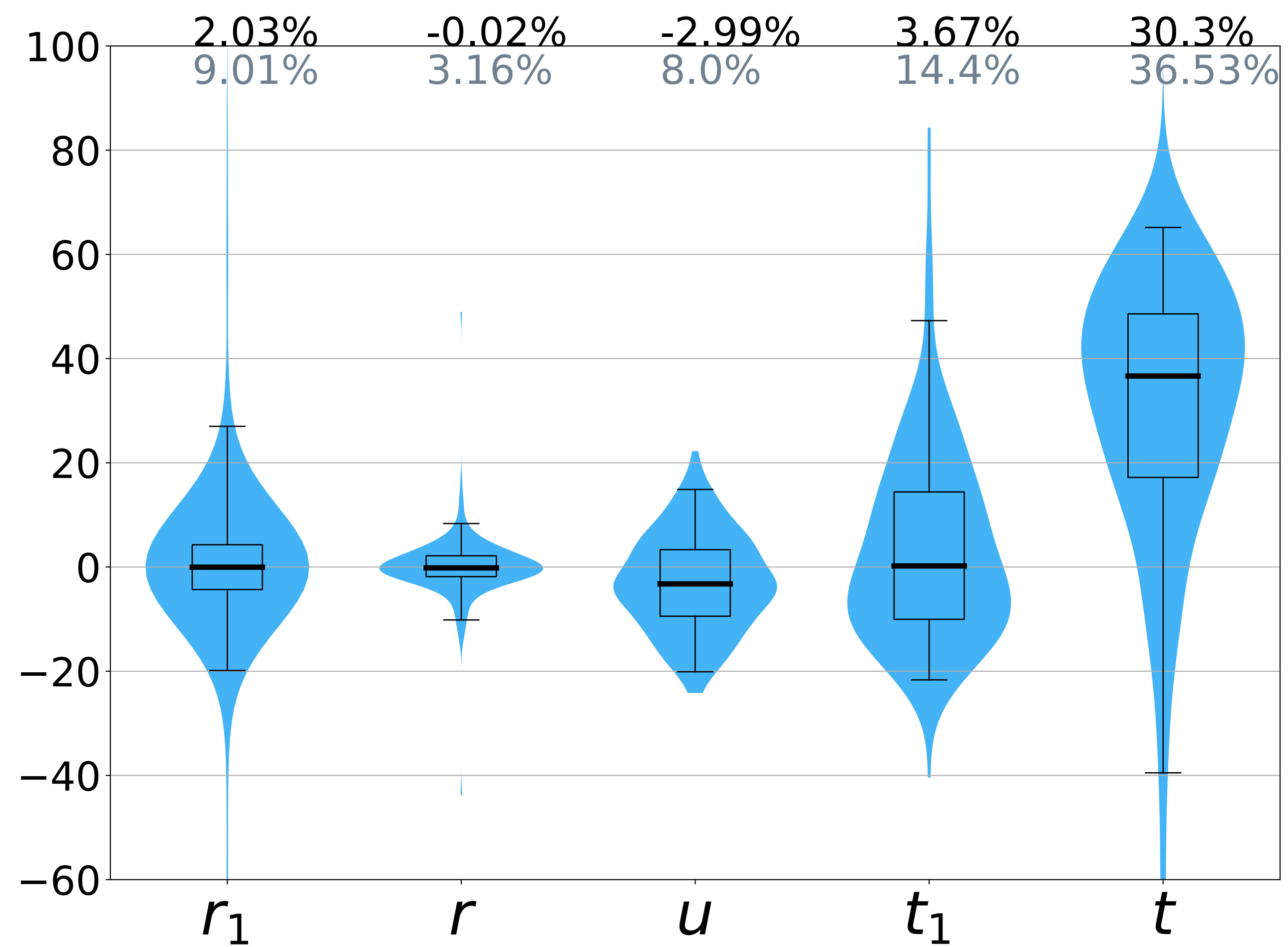**b**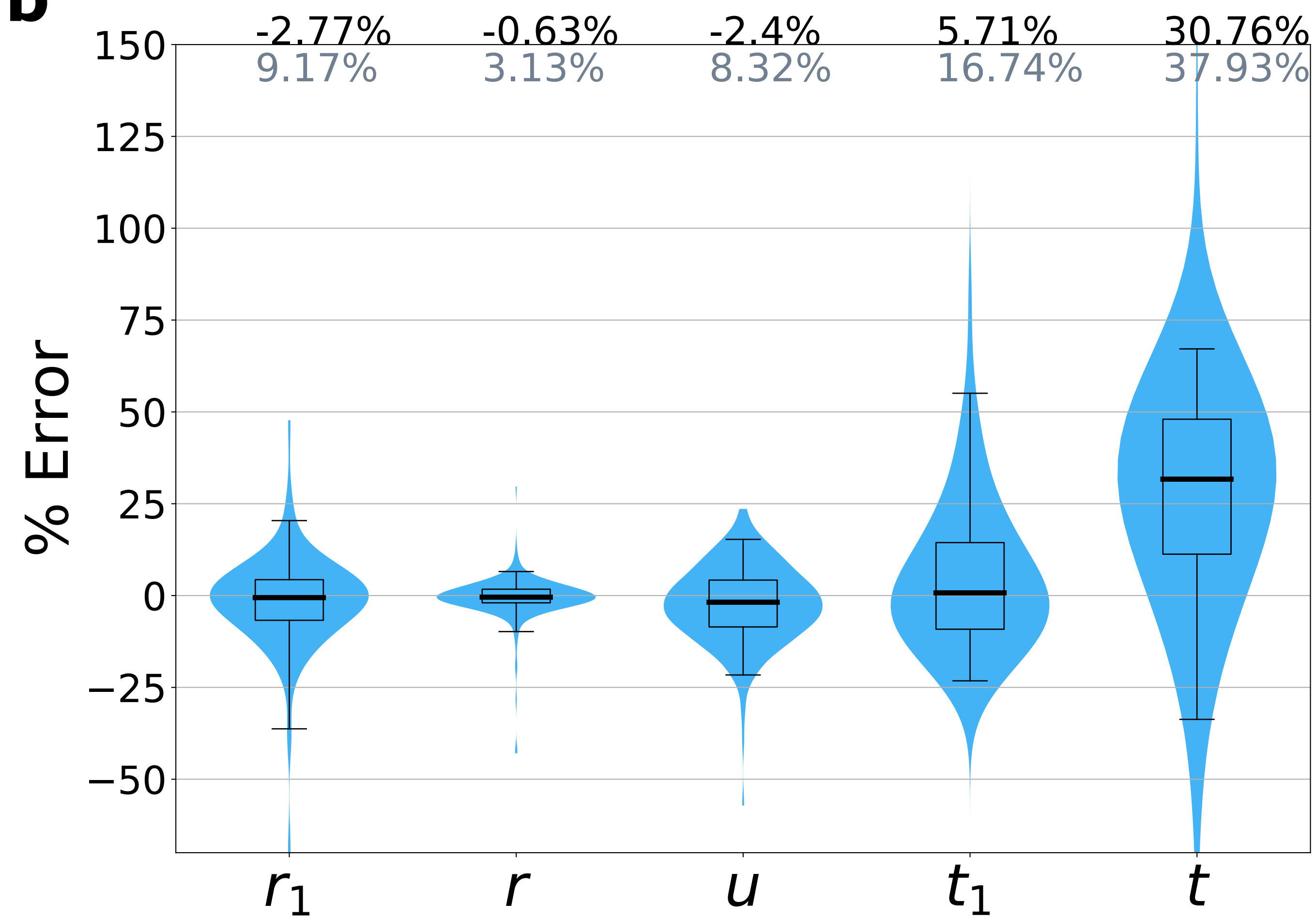**c**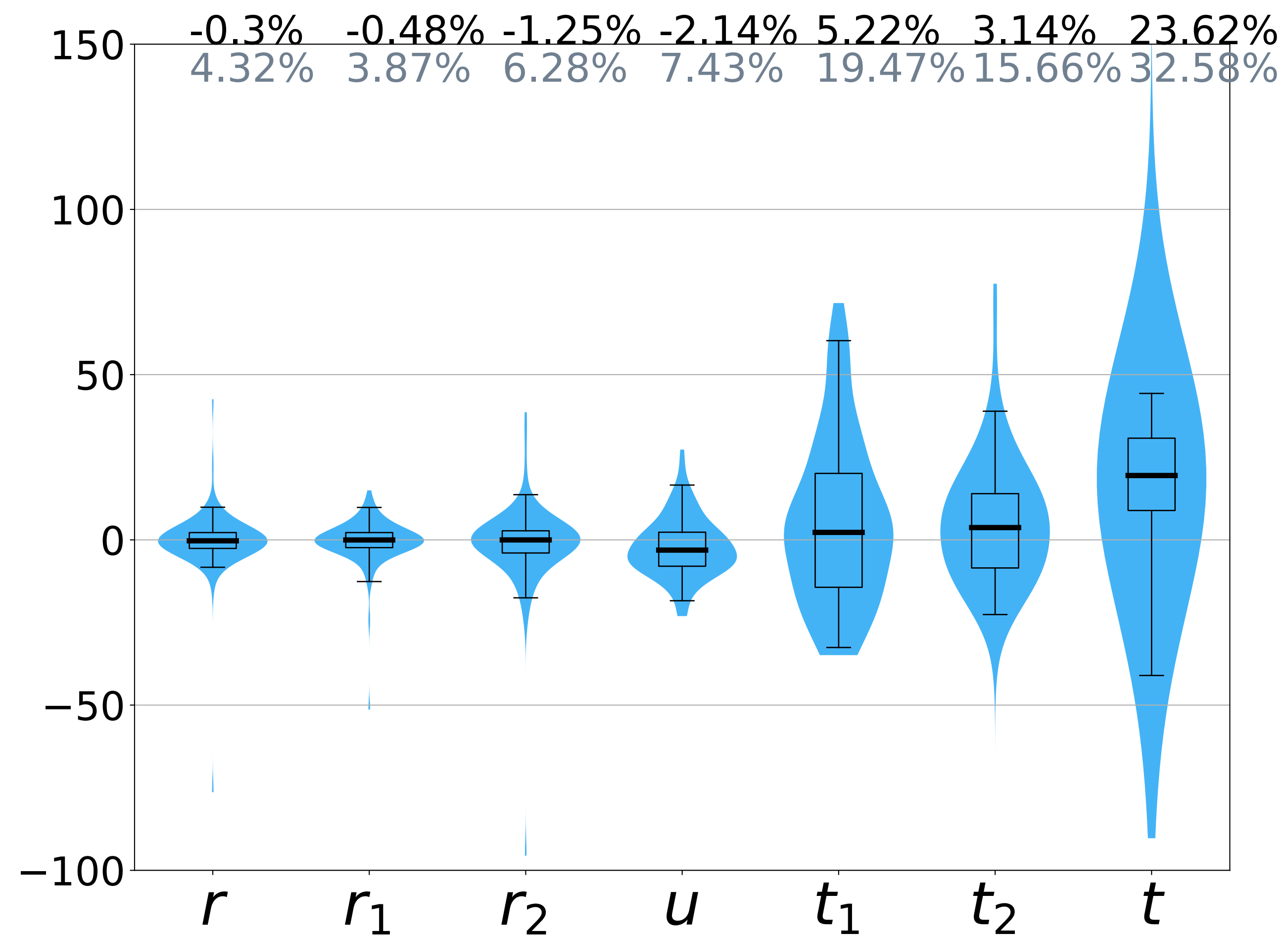**d**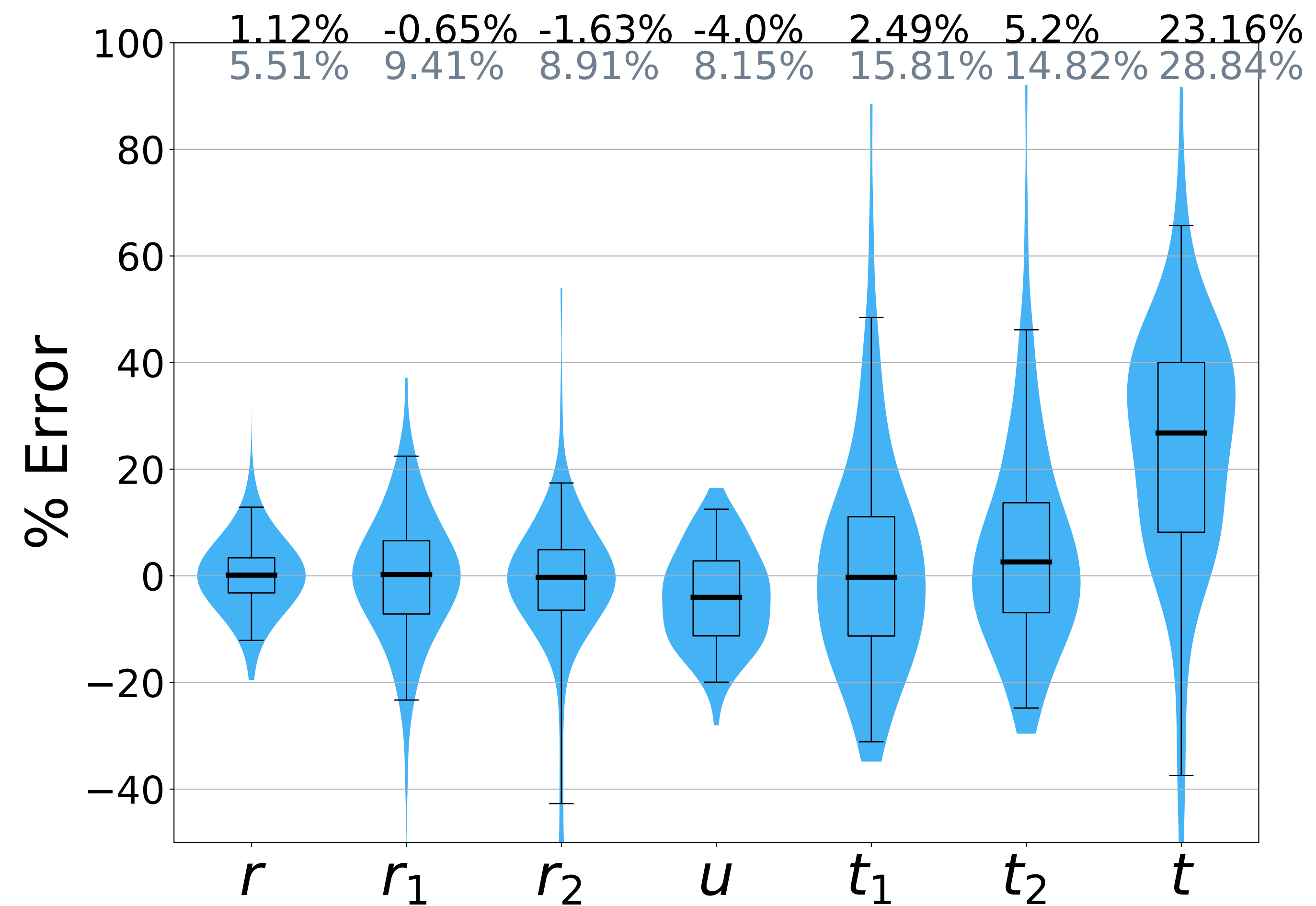

Supplement: S2 Fig — Accuracy of parameter inferences for surviving Monte Carlo simulation runs of slow-growing tumor for (a) single subclone with mutation rate u = 1, (b) single subclone with u = 5, (c) two nested subclones with u = 1, and (d) two sibling subclones with u = 1. Mean percent error (MPEs) are the black numbers above the plots, and mean absolute percent errors (MAPEs) are the grey numbers below the MPEs. Boxes contain 25th-75th quartiles, with median indicated by thick horizontal black line. Whiskers of boxplots indicate 2.5 and 97.5 percentiles. Violins are smoothed density estimates of the percent error data points. Complete parameter values and number of runs are included in S1 Table. (PDF) [file pcbi.1010677.s002.pdf]

**a**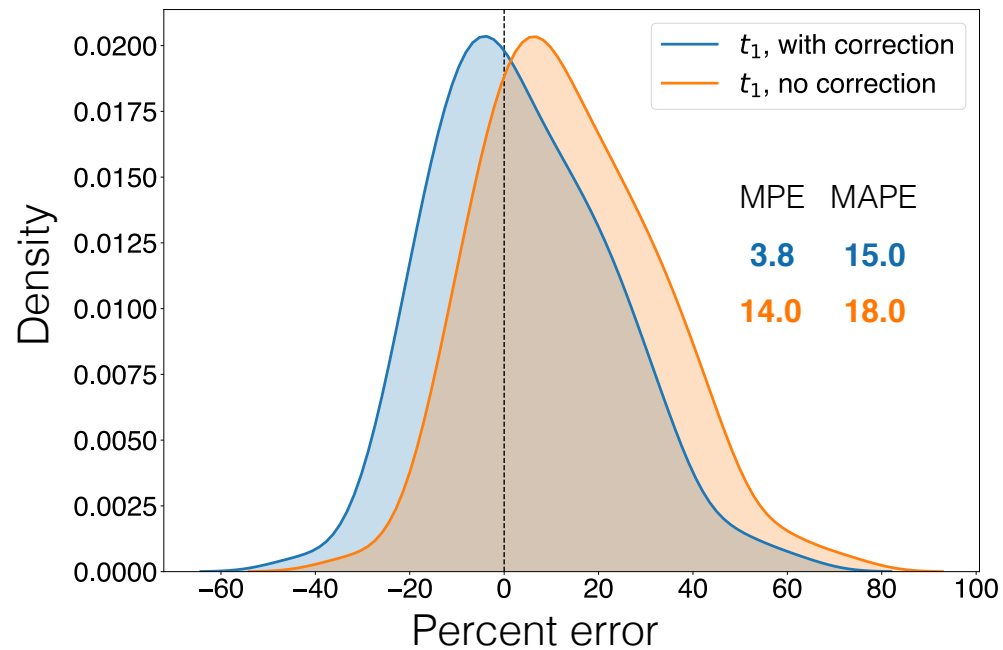**b**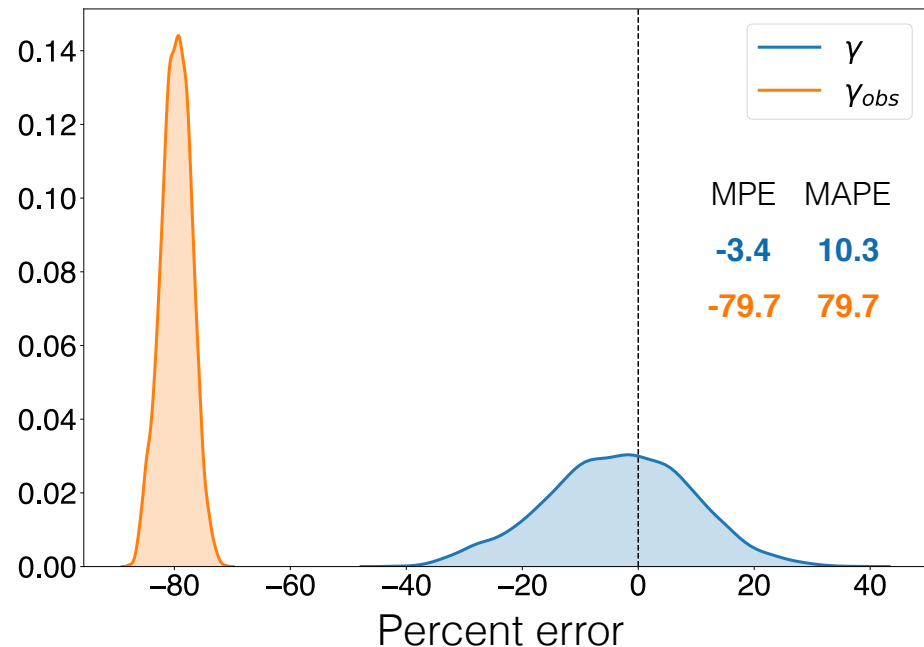

Supplement: S3 Fig — (a) We compare percent errors of parameter estimates for time from tumor initiating until appearance of a driver subclone, t1, with and without the correction for passengers that occur after the driver and reach fixation in the driver population (Eq (6), main text). Errors for estimate with correction are shown in blue, and for estimate without correction (Eq (5), main text) in orange. Errors are plotted as a kernel density estimate for Monte Carlo simulations of fast-growing tumor with mutation rate u = 1. Mean percent errors (MPEs) and mean absolute percent errors (MAPEs) are listed. (b) The percent errors for the observed (orange) and corrected (blue) number of subclonal mutations between frequencies f1 and f2, γ, (Eq (7), main text) are plotted as kernel density estimates. Observed mutations are those that passed post-processing, i.e. those that have more than L = 2 mutant reads. True mutation frequencies were generated from 135 surviving runs of a Monte Carlo simulation of a fast-growing tumor with mutation rate u = 1, from which sequencing reads were simulated with 100x average coverage (see Materials and methods). Percent errors are calculated relative to the true γ measured from the true mutation frequencies. (PDF) [file pcbi.1010677.s003.pdf]

**a**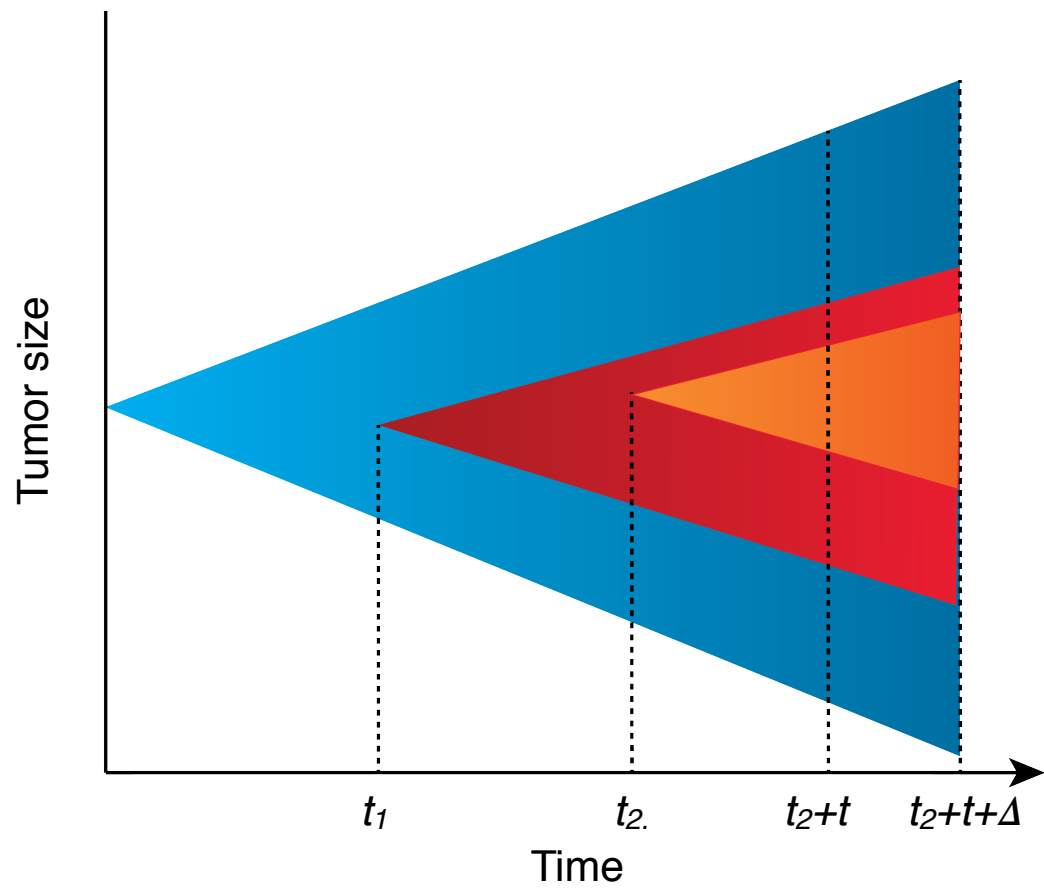**b**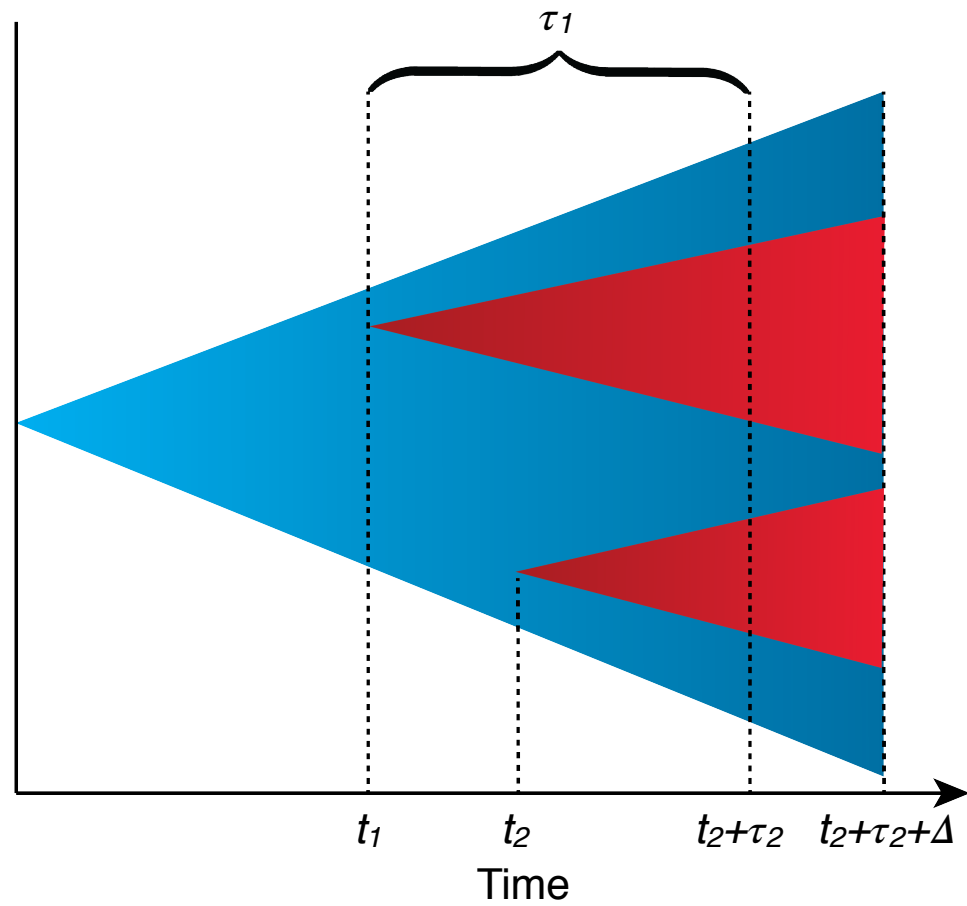

Supplement: S4 Fig — (a) Two nested driver subclones. Initiated tumor (type-0) cells in blue, cells with driver 1 (type-1) in red, and cells with both drivers (type-2) in orange. A driver mutation occurs in a type-0 cell at t1. A second driver mutation occurs in a type-1 cell at t1+t2′. Tumor is bulk sequenced at t1+t2′+t and t1+t2′+t+Δ. (b) Two sibling driver subclones. Type-0 cells (in blue). A driver mutation occurs in a type-0 cell at t1. A second driver mutation occurs in a different type-0 cell at t2. Tumor is bulk sequenced at t1 + τ1 (or, equivalently t2 + τ2) and t1 + τ1 + Δ (equivalently t2 + τ2 + Δ). (PDF) [file pcbi.1010677.s004.pdf]

**a**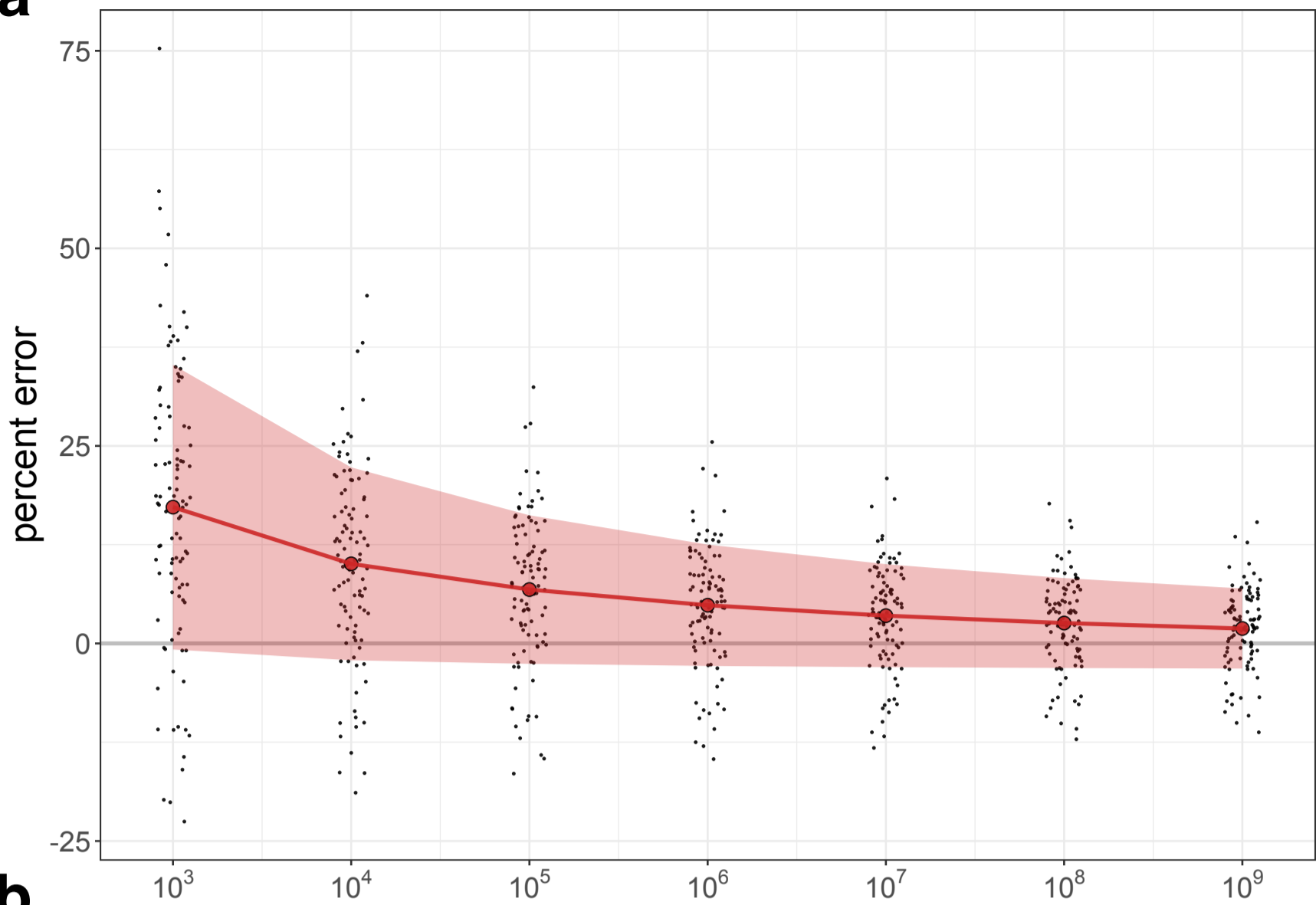**b**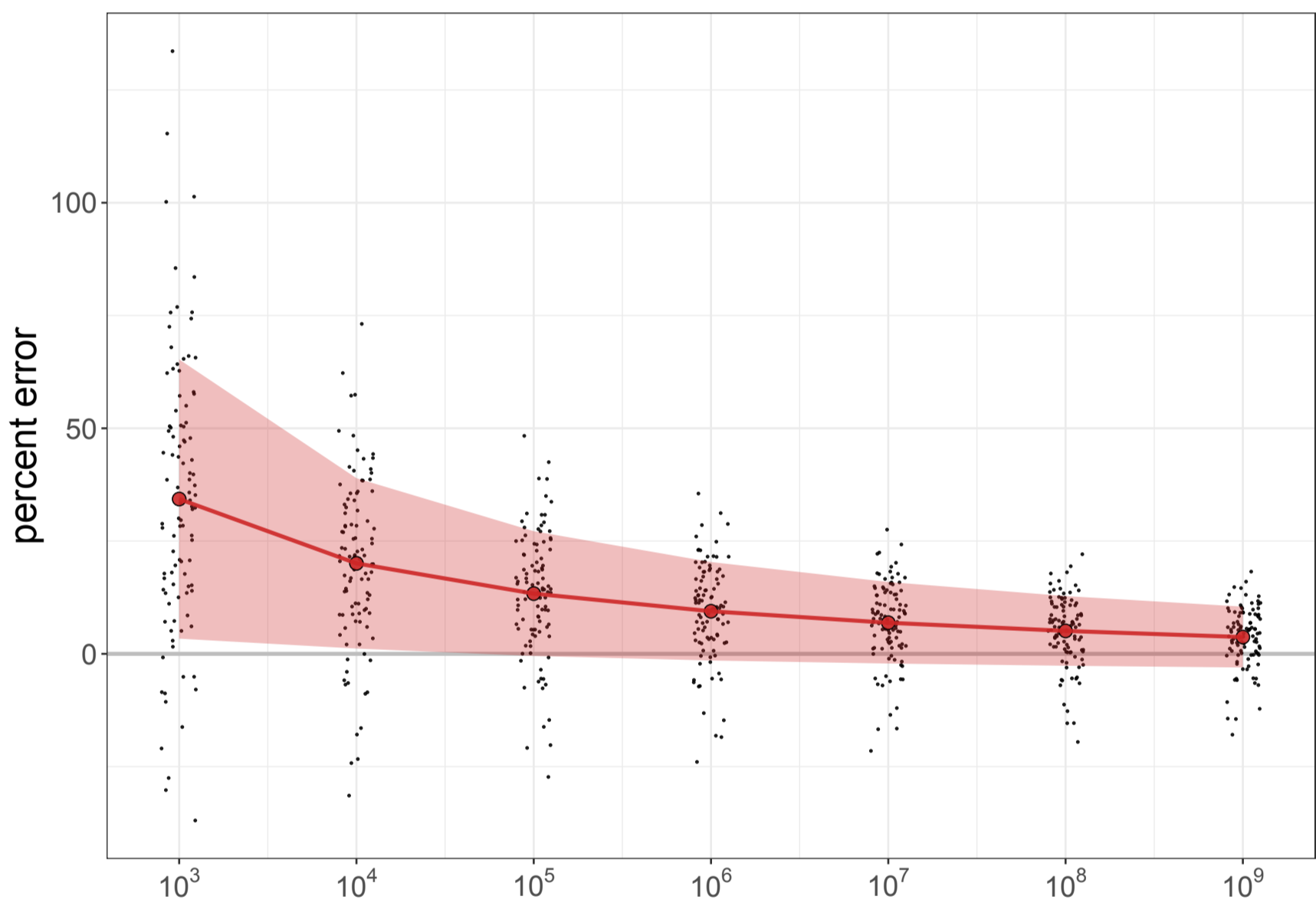**c**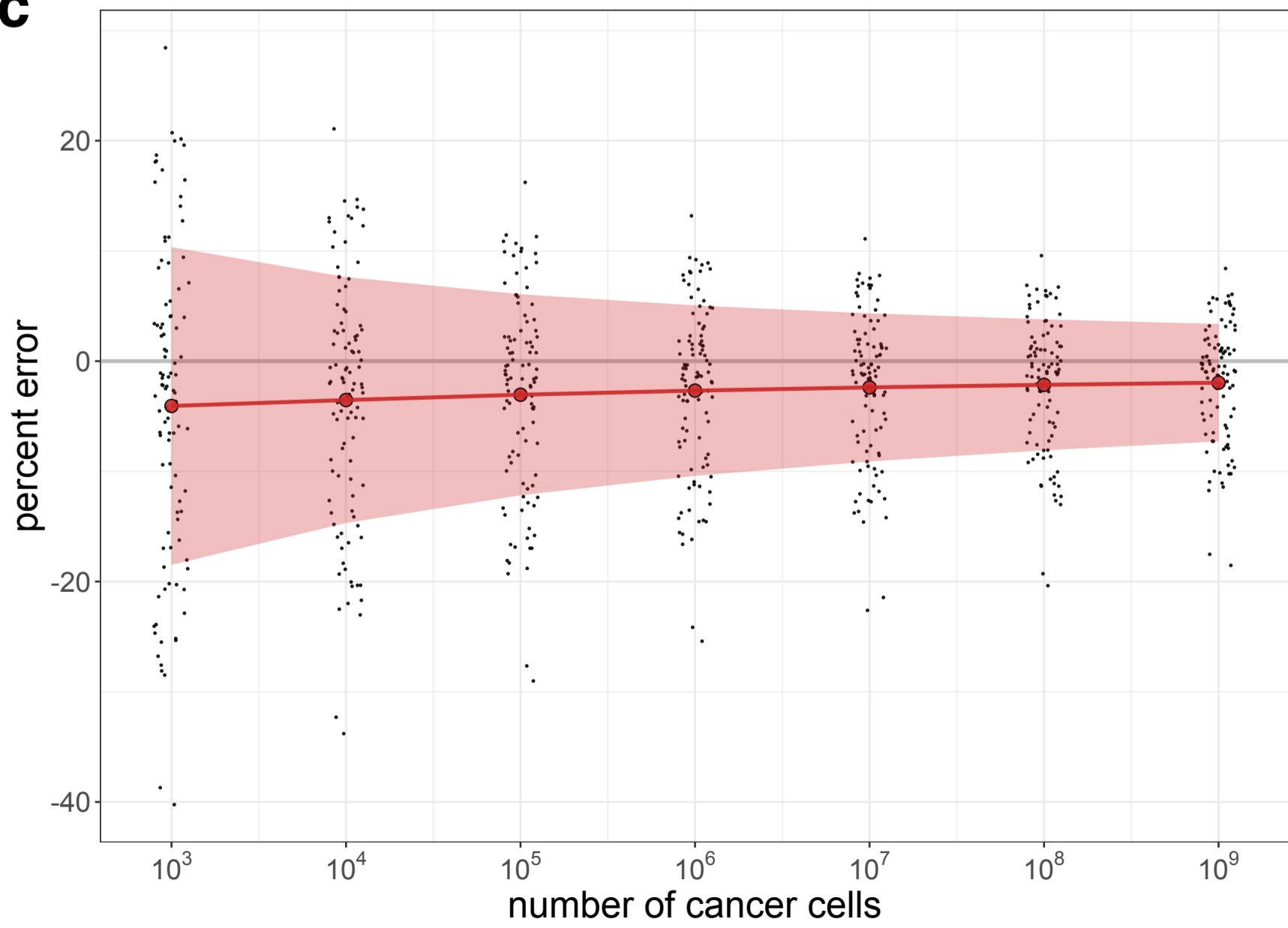

Supplement: S5 Fig — A Monte Carlo simulation of a birth-death process was performed for (a) fast-growing, (b) slow-growing, and (c) no cell death parameter regimes. For each of the 100 surviving simulated tumors, the percent error of the t estimate (Eq (3)) was calculated when the tumor first reached the specified tumor sizes. Means are indicated by red points and lines, ± one standard deviation is shown by the red region, and individual data points for each simulation run are shown as the grey points (with horizontal jitter for visibility). (PDF) [file pcbi.1010677.s005.pdf]
