# Supplementary material for: Inferring parameters of cancer evolution in chronic lymphocytic leukemia
Source: PLoS Comput Biol. 2022 Nov 4;18(11):e1010677. doi: 10.1371/journal.pcbi.1010677 (PMC9668150; doi:10.1371/journal.pcbi.1010677)
Supplement: S1 Methods — (PDF) [file pcbi.1010677.s007.pdf]

S1 Methods. Unbiasedness of growth rate.

Denote our estimator for growth rate of the type-i clone $\widehat{r}_i$, with true parameter value r_i. The type-i population has size $X_i(t)$ at time t. Then,

$$
\begin{aligned}
bias(\widehat{r}_i) &= \mathbb{E}(\widehat{r}_i) - r_i && (1)\\
&= \frac{1}{\Delta}\mathbb{E}\log\Big(\frac{X_i(t_{dx}+\Delta)}{X_i(t_{dx})}\Big) - r_i && (2)\\
&\approx \frac{1}{\Delta}\Big(r_i\Delta + \mathbb{E}\log\ U - \mathbb{E}\log V\Big) - r_i && (3)\\
&= \frac{1}{\Delta}(r_i\Delta + (\gamma + \log r_i) - (\gamma + \log r_i)) - r_i && (4)\\
&= 0 && (5)
\end{aligned}
$$

where U and V are i.i.d. $\mathrm{Exp}(r_i/b_i)$, and t_{dx} is the time of diagnosis.
